# Rethinking Neuroprotection in Severe Traumatic Brain Injury: Toward Bedside Neuroprotection

**DOI:** 10.3389/fneur.2017.00354

**Published:** 2017-07-24

**Authors:** Tommaso Zoerle, Marco Carbonara, Elisa R. Zanier, Fabrizio Ortolano, Giulio Bertani, Sandra Magnoni, Nino Stocchetti

**Affiliations:** ^1^Fondazione IRCCS Ca’ Granda—Ospedale Maggiore Policlinico, Department of Anesthesia and Critical Care, Neuroscience Intensive Care Unit, Milan, Italy; ^2^Department of Neuroscience, IRCCS – Istituto di Ricerche Farmacologiche Mario Negri, Milano, Italy; ^3^Fondazione IRCCS Ca’ Granda—Ospedale Maggiore Policlinico, Unit of Neurosurgery, Milan, Italy; ^4^Department of Pathophysiology and Transplants, University of Milan, Milan, Italy

**Keywords:** traumatic brain injury, neuroprotection, animal models, intensive care unit, multimodal monitoring

## Abstract

Neuroprotection after traumatic brain injury (TBI) is an important goal pursued strenuously in the last 30 years. The acute cerebral injury triggers a cascade of biochemical events that may worsen the integrity, function, and connectivity of the brain cells and decrease the chance of functional recovery. A number of molecules acting against this deleterious cascade have been tested in the experimental setting, often with preliminary encouraging results. Unfortunately, clinical trials using those candidate neuroprotectants molecules have consistently produced disappointing results, highlighting the necessity of improving the research standards. Despite repeated failures in pharmacological neuroprotection, TBI treatment in neurointensive care units has achieved outcome improvement. It is likely that intensive treatment has contributed to this progress offering a different kind of neuroprotection, based on a careful prevention and limitations of intracranial and systemic threats. The natural course of acute brain damage, in fact, is often complicated by additional adverse events, like the development of intracranial hypertension, brain hypoxia, or hypoperfusion. All these events may lead to additional brain damage and worsen outcome. An approach designed for early identification and prompt correction of insults may, therefore, limit brain damage and improve results.

## Panel: Key Points for Improving Preclinical Research

- Evaluation of drug effects in animal models considering different:
  ○ Types and severity of lesion
  ○ Species
  ○ Age, sex, comorbidities
- Assessment of early and late functional and histological outcomes
- Clinically relevant therapeutic window
- Well-elucidated mechanism of action and pharmacokinetics of the candidate compound
- Replication of the effect in different independent laboratories

## Introduction

Progress in neurosurgery, neuroradiology, and critical care medicine in the last 50 years (1) contributed to the drops of 9% per decade in traumatic brain injury (TBI) mortality among hospitalized patients between 1970 and 1990, and it has been stable since (2). However, TBI remains a major cause of mortality and morbidity: approximately 57,000 deaths related to TBI occur in the European Union every year (3). Moreover, the increasing proportion of survivors includes many with neurological disabilities and poor quality of life (4); it was estimated that 7.7 million patients live with TBI-related disabilities in Europe (5). For all these reasons, neuroprotective strategies could provide immense benefits.

Even if there is no broadly accepted definition, neuroprotection in TBI can be considered as the body of interventions aimed at improving the patient’s outcome, and preserving and restoring the integrity, function, and connectivity of the brain cells not irremediably damaged by the initial injury. While the primary injury at the moment of the impact (including hemorrhage, laceration, contusion, and primary axotomy) is not amenable to medical treatment, the complex cascade of molecular and cellular events (secondary injury) that follows the original damage can aggravate the initial harm. This cascade reduces the chances of functional recovery but could, at least theoretically, be counteracted (6, 7).

The first section of this paper discusses attempts to limit the progression of injury, focusing on preclinical research, and translational medicine. In the second section, we describe therapeutic interventions based on multimodal brain monitoring that could reduce the extent of additional insults to the injured brain.

## Neuroprotection in Preclinical Research and Translational Medicine

Traumatic brain injury is the result of an external force applied to the head (8) which, depending on the energy and site of the impact, can result in a number of different lesions commonly referred to as primary injury. Contusions, lacerations, epidural hematomas, subdural hematomas, subarachnoid hemorrhage, and axonal injury may be seen in TBI patients, based on the different mechanisms of the injury (direct impact, acceleration and deceleration forces, penetrating object, explosion blast waves), singly or in combination.

Beyond the brain tissue disruption at the moment of the impact, a broad spectrum of secondary events is triggered by the initial biomechanical injury. This include acute, subacute, and chronic events that all contribute to cell death and/or degeneration and are referred to as secondary brain injury (9). Briefly, alteration in ionic permeability and release of excitatory neurotransmitters (especially glutamate) propagate damage through energy failure and free radicals overload. Spreading depolarization is thought to be linked to this excessive release of glutamate. Cellular permeability is altered and increases calcium influx; this causes mitochondrial dysfunction, priming further energy defects, and apoptosis. Neurons ultimately may die through necrotic and apoptotic processes; autophagy is believed to play a role as well. Damaged axons may further fall prey to secondary axotomy and demyelination. Trauma directly affects the blood–brain barrier, with increased permeability causing protein-rich edema, and activation of a pro-inflammatory state. Inflammation, also promoted by resident microglia, has a dual action, spreading damage and, at the same time, promoting neurorestorative processes. This complex series of events starts immediately (seconds or minutes) after trauma but may last for weeks or months, especially inflammation (4). The contributions of each of these pathways to the secondary brain injury vary depending on the specific TBI lesion; for example, inflammation-mediated brain injury seems predominant in contusion while calcium-mediated injury predominates in diffuse axonal injury (10).

Several TBI models reproduce specific types of lesion in homogeneous groups of animals. Based on the distinct force applied, they can be used to investigate the components of the primary and secondary injury in time and space. Each model has specific advantages and limitations (9). None of them can be considered ideal, but together they lead to an understanding of mechanisms contributing to cell death or dysfunction after TBI. Consequently, experimental models have allowed the identification of therapeutic targets and the study of a wide spectrum of neuroprotective molecules including drugs aiming at specific targets (such as calcium-antagonists, NMDA-antagonists, free radical scavengers, bradykinin antagonists) and also drugs targeting multiple/pleiotropic mechanisms (such as anti-inflammatory steroids, erythropoietin, progesterone) (11).

Despite promising results in preclinical settings, these pharmacological neuroprotective compounds have proved disappointing in human studies. In the last three decades, more than 20 neuroprotective agents have been tested in clinical trials (11) without proof of significant outcome improvement. A recent overview of more than 10 “robust” studies (multicenter, including more than 100 patients, with an appropriate design and a low risk of bias) enrolling more than 15,000 patients confirms their failure to demonstrate any positive result (12).

This translational failure may have numerous reasons, both in the clinical and in the preclinical settings.

Subsequent reappraisals concerning the study design and conduction of clinical trials have been published (11, 13, 14). These analyses identified a number of possible critical factors that may have counteracted the neuroprotective potential of the compounds, for instance:

– the extent of side effects of the drugs and the occurrence of serious systemic complications
– the small sample size with inadequate statistical power
– the enrollment of patients too severe or too mild to detect any benefit
– the use of outcome scales insensitive to important consequence of brain injury
– the high heterogeneity of TBI population without the use of statistical tools for important covariate adjustment
– the inter-center variability in clinical care and clinical outcome.

To overcome this long list of limitations inherent to randomized clinical trials an alternative approach, devoted to Comparative Effectiveness Research, is currently ongoing. An example is the Collaborative European NeuroTrauma Effectiveness Research in TBI (CENTER-TBI^1^), a large scale international project aiming at collection of demographic, clinical, imaging, genetic, and proteomic data from 5,400 TBI patients (15). The main objectives are to improve TBI characterization and classification and to identify the best clinical care, using comparative effectiveness research approach. CENTER-TBI and other similar projects running in Europe and North America, coordinated in the International Initiative for Traumatic Brain Injury Research (InTBIR^2^) are expected to provide high quality data, and rigorous statistical analysis, for improving care and outcome in TBI.

There is also the possibility that the preclinical findings were weaker than expected. First, there are limitations in the experimental models used, which exploit a single traumatic mechanism, such as direct impact or blast waves. However, no single mechanism can reproduce the wide pathophysiological and epidemiological heterogeneity of TBI—a very complex disorder. This means that a therapeutic effect detected in a homogeneous animal population exposed to a single type of injury may well not be generalizable to the human TBI population. Second, the quality of some experimental studies is variable (16, 17), and there is the risk of stressing positive results. It is recognized that blind assessment of the effect, animal randomization, and other indicators of quality are inversely related to the effect size in several published studies on brain injury (18). Third, the papers with negative results are less likely to be accepted for publication. This bias skews the literature and makes any thorough evaluation of treatment effects difficult or impossible (16–18).

In recent years, new approaches and research strategies have been proposed (19–21) to overcome these obstacles (see Panel). Operation Brain Trauma Therapy (21) moves in this direction. OBTT is a consortium of established preclinical TBI investigators supported by the US Army. It aims at identifying promising acute therapies for TBI, testing their efficacy across different animal models and laboratories through rigorous neurological examinations, motor and cognitive tests, brain and lesion volume measures, and biomarkers (22). OBTT recently reported that the neuroprotective effect of four (out of five) potential treatments, rigorously re-tested, was weaker than previously indicated in the literature (22). Those negative findings highlight the need for improving research standards in both preclinical and clinical research.

## Neuroprotection at the Bedside

Repeated failures of pharmacological neuroprotective trials have blunted the enthusiasm for potential new wonder drugs. The number of industry-sponsored studies has markedly dropped, with eight new trials started in 1995–1999 but only one from 2005 to 2009 (13). Interestingly, failures occurred in the decades (1980–2010) during which the fundaments of neurocritical care were established and specialized care for severe brain injury emerged as a discipline.

The first textbook on “Neurological and Neurosurgical Intensive Care” was published by Alan Ropper and Sean Kennedy in 1983 (23). In 1995, the Society of Critical Care Medicine established a neuroscience section; in 2002, the NeuroCritical Care Society was founded. Growing interest in acute brain injury led to a pragmatic approach toward neuroprotection. While awaiting revolutionary pharmacological interventions, it became evident that additional, second insults after initial injury were frequent, and could be prevented and/or minimized in clinical practice. The hypothesis was that clear identification and correction of aggravating factors such as arterial hypotension could reduce the total burden imposed by TBI on the central nervous system and could consequently improve outcomes.

In this section, we describe some aspects of current ICU practice as part of a comprehensive strategy for minimizing insults to the injured brain and restoring brain homeostasis.

### Early Phases after Brain Trauma: Hypoxia and Hypotension

Hypoxia (defined as arterial oxygen tension less than 60 mmHg or peripheral saturation of oxygen less than 90%) and hypotension (defined as systolic arterial blood pressure less than 90 mmHg) (24) in the early phases after TBI are frequent and dangerous insults (24–26). They are fundamental predictors of bad outcome (27, 28). Hypoxia may have multiple causes: direct traumatic pulmonary damage (contusion, pneumothorax, hemothorax), altered gas exchange (shunt, leakage because of increased capillary permeability), and lack of airway protection due to impaired consciousness. Hypotension is most frequently caused by massive hemorrhage or cardiac tamponade.

**Figure 1:**
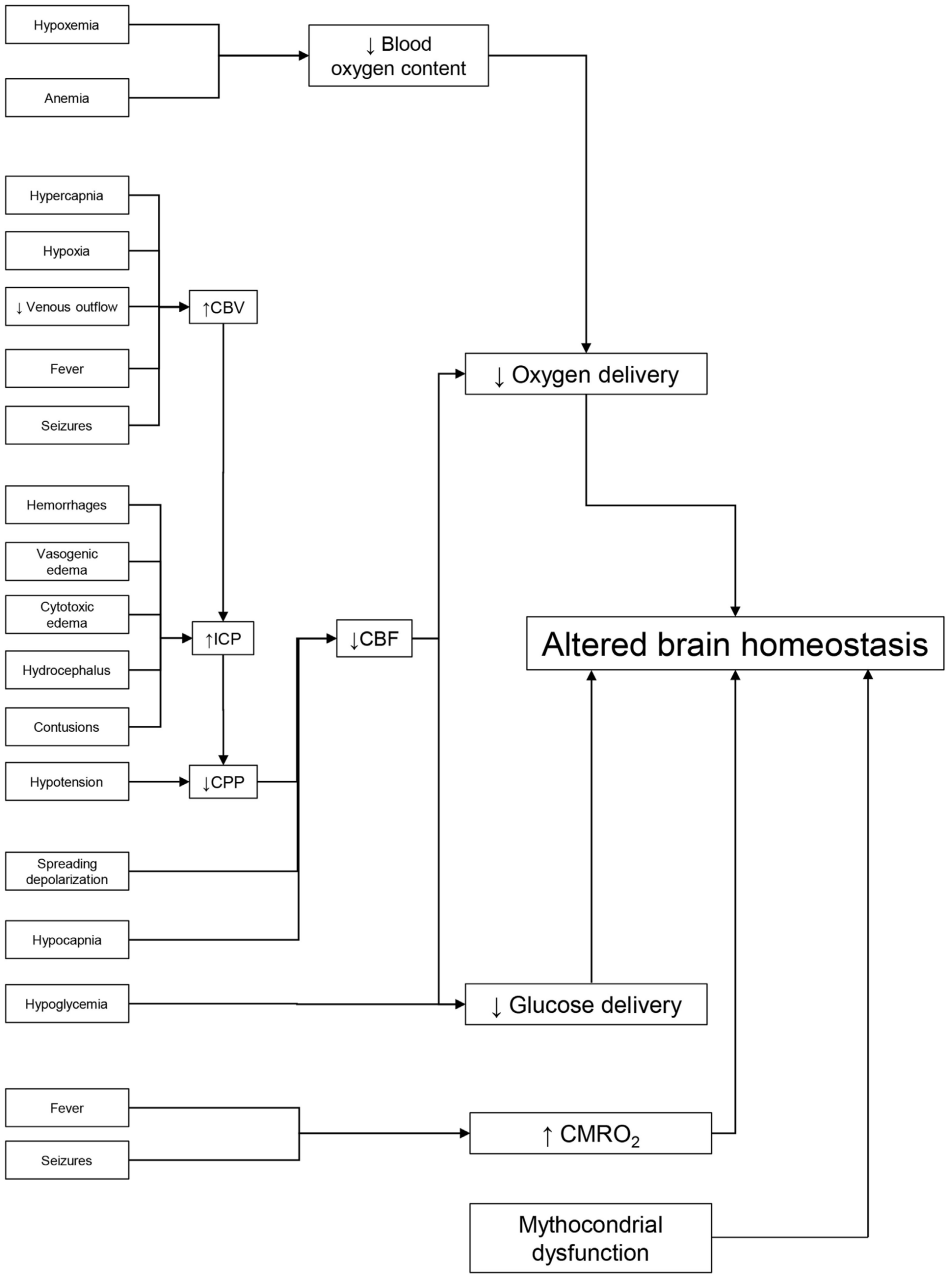
Dealing with potential brain insults at the bedside. Preservation of brain homeostasis requires careful detection of multiple threats, listed on the left side of the figure. Reduced delivery of metabolic substrate and/or increased cerebral metabolic rate of oxygen (CMRO_2_) are the common pathophysiological mechanisms that may alter brain homeostasis. The key elements in oxygen delivery are blood oxygen content and cerebral blood flow (CBF). The first may be limited by hypoxia (secondary to respiratory failure) or by low hemoglobin. Continuous monitoring of CBF in the ICU is difficult but it can be estimated from cerebral perfusion pressure (CPP). Arterial hypotension and/or elevated intracranial pressure (ICP) [secondary to increased cerebral blood volume (CBV), hematomas, contusion, hydrocephalus, and edema] can reduce CBF. Cerebral vasoconstriction secondary to hypocapnia and spreading depolarization can also limit CBF. Glucose delivery is guaranteed by CBF and blood glucose levels. Factors limiting CBF and hypoglycemia (common during intensive insulin therapy) can reduce its supply. Seizures and fever are common causes of high CMRO_2_ in the acute phase of traumatic brain injury. By leading to cerebral vasodilatation, they can raise CBV and ICP, lower CPP, and limit CBF and substrate delivery. Unfortunately, preserving the delivery of oxygen and glucose may not be enough to maintain cerebral homeostasis if their utilization is impaired by mitochondrial dysfunction.

Hypoxia and hypotension may worsen outcomes through two fundamental mechanisms: they may be associated with severe extracranial lesions, such as irreversible shock, which on its own can worsen mortality. Then too, they may amplify the initial damage by impairing oxygen and glucose delivery to an already compromised brain (Figure 1).

Correction of hypoxia and hypotension with prompt airway management, support ventilation, and fluid resuscitation is mandated by international guidelines (29, 30). Airway protection with tracheal intubation, which has been debated in the past (31), is recommended in all patients suffering severe TBI (32). Hypotension must always be avoided and corrected with isotonic fluids and, when necessary, vasopressors. The long-standing debate on hypertonic fluid is still ongoing, with no evidence of superiority. On the other hand, albumin should be avoided. A recent re-analysis of the SAFE data (33) in the subgroup of TBI patients showed albumin infusion was directly related to higher intracranial pressure (ICP) and worse outcome (34). Blood components should be supplied, to optimize oxygen delivery with an adequate hemoglobin level and restore coagulation in hemorrhagic patients (35).

While no study has quantified the protective effect of careful avoidance of hypoxia and hypotension, the extent of additional damage they cause has been well documented in experimental conditions (36–38). A schematic revision of Traumatic Coma Data Bank findings on 717 patients indicated mortality around 25%, in the absence of hypoxia and hypotension; this increased threefold for patients suffering hypoxia and hypotension (24). It seems likely, therefore, that preserving the injured brain from additional hypoxic–hypotensive insults could be beneficial.

### ICP—Cerebral Perfusion Pressure (CPP)

A large amount of observational data (39–42) confirms the association between high ICP and unfavorable outcome, and particularly with increased mortality. High ICP may directly cause brainstem compression and distortion, which explains its relationship with mortality (43). It may also cause a critical reduction of CPP, leading to brain ischemia (Figure 1).

A recent South-American trial (44) on severe TBI, using a treatment strategy based on ICP monitoring compared with a clinical and CT-based strategy, failed to show better outcomes in the ICP group; nevertheless, the value of ICP monitoring still stands (45). Recent guidelines (46) have incorporated this trial, but still suggest ICP monitoring for reducing early mortality after TBI.

Cerebral perfusion pressure, calculated as mean arterial pressure minus ICP, is vital to perfuse the brain because it is the driving force for cerebral blood flow (CBF). The accepted threshold is commonly set at 60 mmHg (46, 47) but a higher threshold might be warranted in patients with impaired autoregulation due to chronic arterial hypertension (48).

The first strategy against dangerous ICP increases and CPP reductions is prompt recognition and removal of expanding intracranial hematomas (43). Reports based on few cases are extremely eloquent and prove that removal of subdural hematomas, while initially causing a destructive reduction of CPP and CBF (49) allowed restoration of cerebral perfusion (50). In this perspective, emergency surgery is an indisputable, effective neuroprotective strategy.

The treatment of increased ICP is based on a graded approach (43), with basic treatment (including sedation and supported ventilation) for all patients; more invasive therapy has to be reserved for more severe cases. Extreme therapies are only recommended for refractory intracranial hypertension, because of troublesome side effects. The concept of dosing therapy and applying more aggressive interventions only to selected patients is also evident from recent trials (51, 52). When highly invasive treatments, such as hypothermia or surgical decompression, were applied to patients with relatively low ICP, the outcome was worse in the treated group.

In clinical practice, careful ICP and CPP monitoring, coupled with tailored therapies, are fundamental neuroprotective tools: a first-line defense against brain stem compression and critical CBF reductions.

### Advanced Intracranial Monitoring: PbtO_2_ and Microdialysis

Inadequate substrate delivery (mainly oxygen and glucose) to the brain is an obvious cause of tissue hypoxia, metabolic disturbances, and potential metabolic crisis. Multimodal monitoring (53) offers the possibility of measuring (and possibly optimizing) several key metabolic parameters in limited volumes of the brain.

Partial brain tension of oxygen (PbtO_2_) can be continuously measured with specific probes, and microdialysis can be used to sample the extracellular concentrations of glucose, lactate, and pyruvate at specified intervals, usually hourly (53, 54). Besides CPP, which may give indirect information on the global CBF driving pressure, these parameters may capture signs of hypoxia, hypoperfusion, and downstream metabolism disturbances. Reduction of PbtO_2_ below a threshold of 20–25 mmHg is associated with worse outcome (55–57).

Microdialysis offers an insight on the metabolic profile of the brain; a normal lactate/pyruvate ratio (LPR) (usually lower than 25) indicates physiologic glucose utilization through the Krebs cycle. The LPR reflects the redox state of the brain (54). When measured together with the extracellular glucose concentration, and possibly with PbtO_2_, different metabolic profiles can be identified. For instance, a low glucose concentration coupled with a high LPR and low PbtO_2_ is consistent with ischemia; mitochondrial dysfunction is suspected when a normal glucose concentration and normal PbtO_2_ are found simultaneously with a high LPR (58). Metabolic disturbances measured by microdialysis are linked with worse outcome after TBI (53, 59). Early or persistent oxidative metabolic dysfunction has been correlated with brain atrophy (60).

These advanced monitoring techniques may measure the adequacy of oxygen and substrate delivery to the brain and identify dangerous alterations. Additional information besides traditional surveillance (based only on ICP and CPP), as provided by PbtO_2_, has given encouraging results (61). Therefore, advanced multimodal monitoring could improve insult detection at the bedside and contribute to better brain protection.

### Brain Electrical Disturbances during the ICU Course after TBI

Traumatic brain lesions, particularly after penetrating injury, are a major risk factor for seizures (62). Guidelines recommend early prophylaxis with phenytoin to prevent seizures in the first week after TBI (46). TBI patients are exposed to other electrical disturbances as well, such as non-convulsive status epilepticus (NCSE) and spreading depolarization. NCSE has been diagnosed with variable incidence in TBI series (63, 64), often regardless of the use of antiepileptic drugs.

Pathological waves of sustained depolarization that propagate through the cerebral gray matter are attracting increasing research interest. They are indicated as spreading depolarization (65) and are associated with microvascular and metabolic alterations. Their exact pathological role and the potential benefit of specific treatments are still under investigation.

Seizures and analogous electrical disturbances (65, 66) demand energy. Uncontrolled hyperactivity of neurons can induce or worsen a metabolic crisis in the injured brain. Therefore, prevention of seizures and appropriate monitoring of electric activity (in selected cases by continuous EEG) can help prevent, or disclose, noteworthy second insults (67), offering additional protection.

### Fever

Hyperthermia is deleterious to the damaged brain (68, 69). It can exacerbate ischemic injury (by increasing the brain’s metabolic demand) and may cause vasodilation of the cerebral vessels. This increases the brain–blood volume and may worsen ICP (70). Ample evidence indicates that fever is dangerous in TBI patients, worsening morbidity, and mortality (71).

While repeated trials have reported that hypothermia offers no benefit (51, 72), it is agreed that hyperthermia is definitely an insult after TBI. Careful temperature monitoring, and treatment of fever, may therefore reduce further brain damage in the acute phase.

## Conclusion

The paradox of neuroprotection in TBI is that, despite a long list of potential neuroprotective agents active under experimental conditions, no compound has demonstrated protection in clinical trials. Analysis of clinical and preclinical trials has identified several gaps and improvements are certainly needed. However, even the most rigorous scrutiny of evidence and the highest research standard, as proposed by OBTT, do not guarantee success: a similar initiative in ischemic stroke (73) led to a negative clinical trial (74).

While awaiting an effective molecule limiting secondary brain injury after trauma, good-quality neurointensive care can provide modest but effective neuroprotection. By monitoring systemic and neurological parameters, intracranial and extracranial threats can be identified. In this way, effective targeted therapies become possible, and the burden of additional insults to the brain might be lightened.

## Author Contributions

TZ, MC, and NS designed the review, assembled a preliminary draft, and incorporated further contributions from each author into subsequent versions. All the authors revised it critically for important intellectual content and approved the final version.

## Conflict of Interest Statement

The authors declare that the research was conducted in the absence of any commercial or financial relationships that could be construed as a potential conflict of interest.

## Footnotes

^1^https://www.center-tbi.eu.

^2^https://intbir.nih.gov/.
